# Relatively drunk: subjective intoxication and estimated health consequences of alcohol consumption are conditional on the presence of less intoxicated individuals, not level of intoxication

**DOI:** 10.1186/1940-0640-7-S1-A29

**Published:** 2012-10-09

**Authors:** Simon Moore, Alex Wood, Gordon Brown, Jonathan Shepherd

**Affiliations:** 1 Violence & Society Research Group, Cardiff University, Cardiff, South Glamorgan, UK; 2 School of Psychological Sciences, University of Manchester, Manchester, UK; 3 Department of Psychology, Warwick University, Coventry, UK

## 

According to the relative rank hypothesis (RRH), the magnitudes of sensory stimuli are estimated relative to their rank among related comparators. This study assessed the RRH in social drinkers and tested whether rank breath alcohol concentration is a better predictor of subjective alcohol risk estimates. The design was a cross-sectional alcometer street survey. Data from 1997 respondents (mean age, 26.96 years; 61.86% male) were used in analyses. Data were collected from five night-time economies characterized by excessive alcohol use. Prospective respondents were randomly approached to complete a short survey, including perceptions of the long-term health effects of their current state of intoxication and their self-perceived drunkenness, with those who consented to participate providing a breathalyzer score. Alcometer score was ranked according to other respondents and by discrete reference groups. Those whose breath alcohol content ranked high in their reference group reported that they felt more drunk and that they perceived the negative health consequences of alcohol misuse as more likely. No effect of breath score or social norm was observed. Our results generalize fundamental psychophysical theory and indicate that attitudes are spontaneous and relative to context; and that manipulating the social context in which alcohol is served—for example, by including more sober individuals—would increase perceived intoxication and increase the perceived likelihood of alcohol-related poor health. This is contrary to assumptions indicating attitudes are stable and informed by perceived social norms or absolute levels of consumption.

